# Preliminary Outcomes of a Computerized CBT/MET Intervention for Depressed Cannabis Users in Psychiatry Care

**DOI:** 10.26828/cannabis.2018.02.004

**Published:** 2018-07-07

**Authors:** Suzette Glasner, Frances Kay-Lambkin, Alan J. Budney, Michael Gitlin, Bruce Kagan, Helene Chokron-Garneau, Alfonso Ang, Alexandra Venegas

**Affiliations:** 1 Department of Psychiatry and Biobehavioral Sciences, University of California, Los Angeles, Los Angeles, CA, USA; 2 School of Nursing, University of California, Los Angeles, Los Angeles, CA, USA; 3 The University of Newcastle; 4 Geisel School of Medicine, Dartmouth University

**Keywords:** Cannabis, treatment, comorbidity, outcome, depression

## Abstract

Although depression is common among cannabis users, there is a paucity of targeted interventions addressing depression and cannabis use disorders concurrently. In the present pilot study, we examine the feasibility, acceptability, and preliminary outcomes of a computer-assisted intervention combining cognitive behavioral therapy (CBT) and motivational enhancement therapy (MET) techniques for adults with comorbid major depressive disorder (MDD) and cannabis use disorder (CUD) presenting for care in a psychiatric setting. Adults with MDD and CUD (N=26) recruited from mental health care settings were enrolled in a 10-week, computer-assisted psychosocial intervention: Self-Help for Alcohol and other Drug Use and Depression (SHADE). Feasibility, acceptability, perceived helpfulness, treatment retention, completion, and clinical outcomes including cannabis use and depression were assessed. Participants found the SHADE intervention to be acceptable and helpful in facilitating action towards their therapeutic goals concerning depression and cannabis use. Treatment completion, achieved by the majority (85%) of participants, was excellent. On average, participants reduced their past 30 day cannabis use from baseline (mean percentage of days using = 69%) to follow-up (M=44%) (t(22)= 2.3, p<0.05; Effect Size= 0.79). Concurrently, they evidenced reductions in depressive symptom severity, from the moderately severe range at baseline to the mild range at follow-up (t(24)=7.3, p<0.001; Effect Size=1.52). Addressing comorbid CUD and MDD using a computer-assisted, evidence-based treatment strategy is feasible in a psychiatric care setting, and may produce improvements in both depressive symptoms and cannabis use.

Cannabis use disorders (CUD) are associated with four times the risk of developing a subsequent depression (Bovasso, 2001; Patton et al., 2002); likewise, high rates of cannabis use have been consistently demonstrated among individuals with mood disorders (Degenhardt, Hall, & Lynskey, 2001; Farrell et al., 2003; Merikangas et al., 1998). Australian data suggest that depression is the most commonly cited condition for which cannabis is used medicinally (56%), with two-thirds reporting the use of cannabis to assist in coping with emotional difficulties (Swift, Gates, & Dillon, 2005). Regardless of the precise etiology, it remains important to consider depression when managing cannabis use, particularly given that depression is the leading cause of disability in the world.

Published reviews indicate robust effects of psychosocial treatments for CUD (Davis et al., 2015; Budney, Roffman, Stephens, & Walker, 2007; McRae, Budney, & Brady, 2003; Nordstrom & Levin, 2007). The great majority of controlled studies have evaluated motivational enhancement therapy (MET), cognitive-behavioral therapy (CBT), and contingency management (CM) interventions, both as standalone and combined treatment approaches. MET combined with CBT outperformed MET alone in a large multi-site study (Copeland, Swift, Roffman, & Stephens, 2001; Babor, 2004), and this approach is considered to be the current state-of-the art psychosocial approach for CUD (Budney et al., 2007). Enhancing dissemination and implementation of CBT and MET using technology is of great importance because, although CBT and MET are highly efficacious, (a) availability is low, as few treatment programs provide CBT and MET, (b) resources needed to train staff and achieve fidelity of treatment delivery are not widely available, and (c) high caseloads and turnover rates increase the difficulty of maintaining quality (McLellan, Carise, & Kleber, 2003; Glasner-Edwards & Rawson, 2010). Likewise, evidence-based psychotherapies for depression have limited accessibility for similar reasons (Andrews, Cuijpers, Craske, McEvoy, & Titov, 2010; Kiluk et al., 2011).

There is an expanding literature on the use of technology-assisted interventions for the treatment of CUD. Systematic reviews and meta-analyses report effect sizes in the range of 0.11–0.19, indicating small but significant effects of technology-assisted treatments on cannabis use outcomes (Tait, Spijkerman, & Riper, 2013; Hoch et al., 2016; Olmos, Tirado-Munoz, Farré, & Torrens, 2017). Likewise, there is a burgeoning literature supporting the use of internet-based interventions in the treatment of mental health conditions such as depression, with therapist-assisted cognitive behavioral therapy (CBT) producing the largest effect sizes, ranging from 0.6–1.9, followed by moderate effect sizes resulting from stand-alone CBT (0.3–0.7) (see Saddichha et al., 2014). Computer-assisted interventions for substance use disorders have largely been tested in primary addiction treatment settings (Carroll et al., 2008), however, these approaches may have the greatest utility in settings where substance use disorders are underdiagnosed, such as in primary mental health or primary care service delivery systems. Providing services to address substance use in such settings is feasible (Madras et al., 2009; Ernst, Miller, & Rollnick, 2007), can reach many more individuals who need treatment, promises better outcomes (Babor et al., 2007; Brousselle, Lamothe, Sylvain, Forro, & Perreault, 2010), and can reduce health care utilization costs (Parthasarathy, Mertens, Moore, & Weisner, 2003; Parthasarathy, Weisner, Hu, & Moore, 2001).

In the present study, we pilot tested a computer-assisted CBT/MET intervention targeting both CUD and major depressive disorder (MDD), Self-Help for Alcohol and other Drug Use and Depression (SHADE), in a primary psychiatric care setting. The efficacy of SHADE in community based samples of adults with comorbid alcohol use disorders or CUD and MDD has been established in two RCTs (Kay-Lambkin, Baker, Lewin, & Carr, 2009; Kay-Lambkin, Baker, Kelly, & Lewin, 2011). In the first trial, cannabis users with MDD (n=43) reported twice the reduction in cannabis use in response to SHADE, relative to those who received therapist-delivered CBT/MET, and approximately five times the reduction as compared to those who received a single brief intervention session (BI). Likewise, SHADE was associated with a greater reduction in depression relative to BI, and equivalent reductions relative to therapist-delivered CBT/MET. These findings were replicated in a larger RCT (n=109 primary cannabis users), with those in SHADE reporting twice the reduction in cannabis use during treatment relative to therapist-delivered CBT/MET (Kay-Lambkin et al., 2011), and those in the control condition slightly increasing cannabis use.

Of note, in the trials conducted by Kay-Lambkin and colleagues, participants were recruited from the community, and thus were treatment-seeking for their cannabis use and MDD. Nevertheless, computer-assisted approaches to treating substance use disorders may have the greatest utility in clinical settings where: (1) substance use may be under-recognized and/or undertreated, and (2) evidence-based addiction treatment is not readily available. Replicating and extending the prior work of Kay-Lambkin and colleagues, our pilot study was designed to establish the feasibility, perceived helpfulness, and preliminary efficacy of the SHADE intervention in a primary psychiatric care setting, in a population comprising adults presenting for treatment of MDD. In this setting, we anticipated variability in participants’ recognition of their problematic cannabis use, and correspondingly, in their motivation to change their use of cannabis.

We hypothesized that individuals with MDD and comorbid CUD presenting for psychiatric care would find the SHADE intervention to be user friendly, helpful in achieving their goals concerning cannabis use and depression management, and participation in the intervention would be associated with reductions in cannabis use and depressive symptom severity.

## METHOD

### Participants

Participants were 26 adults with CUD and MDD. The study was approved by the UCLA Institutional Review Board. Participants were recruited from outpatient psychiatric clinics at the UCLA medical center and campus Counseling and Psychological Services center through flyers, clinician referrals, and word of mouth. Flyers indicated that the study intervention addressed both depression and cannabis use. A trained research assistant screened all potential participants for eligibility by phone using a brief script. To be eligible for the study, participants were required to: (1) be > 18 years old; (2) have a DSM-5 diagnosis of CUD and lifetime MDD (assessed using the Psychiatric Research Interview for Substance and Mental Disorders [PRISM]; Hasin et al., 1996); (3) be able to read and understand English at or above the 6th grade level; (4) report cannabis use on at least 40 of the past 90 days (assessed using the timeline follow-back); (5) score 9 or higher on the Current Patient Health Questionnaire-9 (PHQ-9), indicating clinically relevant depressive symptom severity; and (6) be on a current antidepressant medication regimen. Additionally, given that participants varied in terms of level of insight concerning problematic cannabis use or CUD, the research assistant explained that while quitting or cutting back cannabis use was not required, participation requires a willingness to examine how depression and cannabis use patterns may relate to one another, and consider changing cannabis use. Individuals were excluded if they: (1) exhibited medical impairment that compromised their safety as a participant; (2) were dependent on alcohol or any other substance from which medical detoxification was required or; (3) had a diagnosis of Schizophrenia or Schizoaffective Disorder. To optimize generalizability, individuals with a diagnosis of Bipolar Disorder were not excluded. After complete description of the study to participants, informed consent was obtained.

Participants agreed to: (1) weekly computerized SHADE intervention sessions to be completed at the clinical research center, followed by a brief check-in with a study clinician for 10 weeks; and (2) in-person assessments weekly and at 1-month follow-up, with $20 compensation for each weekly assessment and $40 for the follow-up.

**Figure 1.**
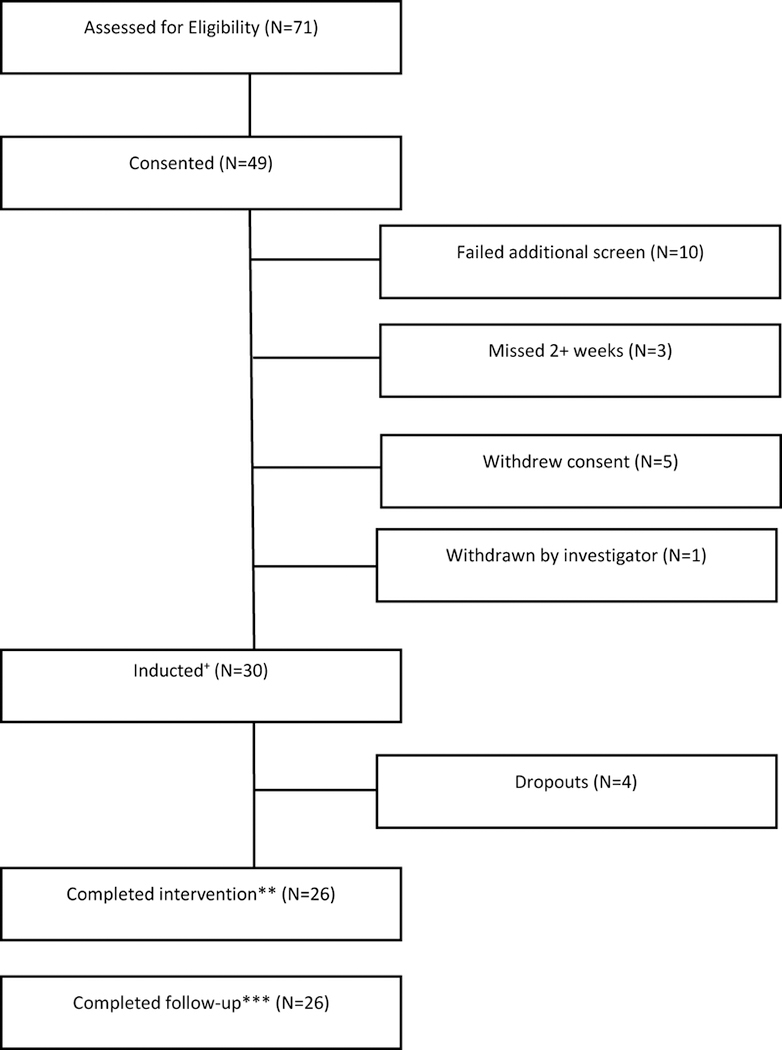
Participant flow throughout the study. +Inducted, received at least 1 SHADE session, **completed week 10 of the intervention, ***completed week 14 follow up

Over the 1-year study period, 71 individuals were screened, of whom 49 consented to participate. Of those, 30 individuals were inducted and 26 individuals completed the SHADE intervention program. Figure 1 depicts the study participant flow.

#### Sample Characteristics

Of the 49 consented participants, 10 failed the additional screen, and 9 were terminated from the study prior to initiating treatment. These 9 participants were terminated for the following reasons: 5 withdrew consent for reasons such as not enough compensation, transportation issues, or no longer being interested in participating, 3 did not return to complete baseline assessments after screening, and one participant was withdrawn by the investigator for acute and severe mental health symptoms that interfered with study participation. There were no statistically significant differences in baseline demographic characteristics, cannabis use frequency, and depressive symptoms between those who were consented and initiated SHADE treatment (n=30) and those who were enrolled but did not initiate (n=9). For those who initiated SHADE treatment, an active protocol to optimize retention included weekly outreach in the form of reminders, and follow-up calls for missed visits via phone, text messaging, and email to optimize treatment completion. Four participants dropped out after initiation of SHADE and were lost to follow up.

The participants were on average, 29 years of age (SD=10.9), with a mean of 12.5 years of education (SD=1.8) (See Table 1). The sample was predominantly female (54%), never married (80%), and Caucasian (73%). On average, participants reported cannabis use on 20 of the past 30 days (SD=11.2) at baseline. Alcohol use in the past 30 days was reported by 61% (n=16) of the sample, with an average of 4.5 days using alcohol (SD=6.5) and 3.8 drinks per drinking day (SD=3.3). Tobacco use in the past 30 days was reported by 31% (n=8) of the sample, with an average of 5.7 days of self-reported use (SD=10.7). In terms of ancillary treatment, all participants were in psychiatric care for MDD and none had received CUD or other substance use disorder treatment in the past 30 days. In the past 90 days, 19% (n=5) reported having attended one or more 12-step self-help meetings, and the majority (80%; n=21) reported having received individual psychotherapy.

### Procedure

#### Design

Eligible participants completed a baseline assessment with a research associate to confirm eligibility and begin study procedures. Once eligibility was confirmed, participants were scheduled for weekly visits over the course of 10 weeks. At each visit, participants completed the weekly computerized SHADE treatment session (Kay-Lambkin, Baker, & Bucci, 2002b; Kay-Lambkin et al., 2009), followed by a brief 10–15-minute check-in with a licensed therapist. Participants also completed a battery of assessments, at each weekly visit, administered by a research associate. Early termination from the study could be a result of missing two consecutive data collection visits or missing two consecutive SHADE sessions.

### Intervention

#### Self Help for Alcohol and Other Drug Use and Depression (SHADE)

SHADE comprises a 10- week, 10-session, computer-assisted CBT/MET intervention for CUD and Major Depressive Disorder (Kay-Lambkin et al., 2011). SHADE begins with a face-to-face brief, tailored motivational intervention session, in which goals around changing cannabis use are explored and established, and the relationship of cannabis use to depression is examined, and individualized feedback is provided. The computerized SHADE modules are subsequently initiated in weeks 2 through 10. The text in SHADE computer modules is at the 6th grade reading level, and has audio accompaniment. At each visit, a research clinician met with the participant for a 10–15-minute ‘check-in’ session, which included: review of homework exercises; development of plans for completing homework; brief suicide risk and mood assessment; and confirmation of the next appointment. A motivational interviewing style is employed in all clinician portions of the intervention, including the brief intervention session and the weekly check-ins.

#### SHADE Content

The SHADE program uses a client-centered approach in that participants choose their therapy goals (e.g., to reduce versus abstain from cannabis use). It also applies CBT strategies, as the program encourages participants to explore the possible relationships between depressive symptoms and cannabis use problems. Motivational interviewing techniques are also a key feature of the intervention; later sessions incorporate discussions regarding making and sustaining changes regarding cannabis use. Session 1 is a brief intervention emphasizing feedback concerning cannabis use, brief advice, and psychoeducation concerning both depression and cannabis use. The remaining 9 computerized sessions include modules on understanding cannabis use patterns, coping with cravings, mood monitoring, managing thoughts about using drugs, problem solving, drug refusal skills, coping with lapses, managing negative moods, assertiveness skills, and coping with life problems. Explicit links between MDD and CUD are made throughout. The programming employs three technological styles. First, computer-assisted instruction presents information, requires active responses to queries designed to assess knowledge acquisition, and evaluates and provides immediate feedback. Second, actors model coping behavior via video-based simulation (e.g., drug refusal skills). Third, interactive exercises and worksheets are utilized to enhance learning and personalize content. Participants use a unique password to access their program via the Internet in a designated clinic office. Prior computer experience is not necessary as the first module provides training.

The master’s level study therapist, who was formally trained prior to this study in CBT and MET, received standardized training on explication, demonstration, and role-playing of the SHADE intervention components, from Dr. Kay-Lambkin, who developed the program. Dr. Kay-Lambkin provided weekly supervision to the study therapist throughout the course of the trial.

### Procedures and Instruments

Trained interviewers conducted assessments at baseline, weekly during treatment, and at discharge and 1-month follow-up. Cannabis Use Disorder was diagnosed at baseline using the PRISM (Hasin, 1996).

#### Cannabis Use

Cannabis use was assessed using urine drug screens and the Timeline Follow Back (TLFB), a calendar assisted structured interview (Sobell & Sobell, 1992) with demonstrated validity in substance treatment samples (Fals-Stewart, O’Farrell, Freitas, McFarlin, & Rutigliano, 2000). The TLFB was used to assess the number of days and daily use frequency in the preceding 30-day period at baseline, weekly during the intervention phase, and 30 days prior to follow-up. Daily use frequency was assessed over each one-hour interval of the day (see Hughes et al., 2014). For instance, if a participant smoked cannabis three times between 10–11, this was recorded as one episode of use. If a participant smoked cannabis at 10 and then at 11, this was recorded as two episodes of use. Abstinence was examined as an exploratory outcome, given the variability in participants’ motivation to reduce versus abstain from cannabis use. To this end, urine specimens were collected at baseline, weeks 1 through 10 and at follow-up and tested using temperature controlled, FDA-approved one-step, test cups (Cliawaived Inc). Creatinine level (<30 ng/ml) was used to assess validity of the specimen, and an invalid specimen prompted requests to provide another specimen within 4–24h. Based on prior studies (Derogatis & Melisaratos, 1983), the primary abstinence outcomes were the number of cannabinoid-negative urine specimens provided during the trial (ranging from 0 to 10) and the longest duration (i.e., weeks) of continuous abstinence.

Cannabis outcome goals were assessed using the Marijuana Outcome Goals questionnaire (Lozano et al., 2006). Participants were asked about their goals with respect to changing their use of cannabis. Answer choices were to “not use marijuana at all” or “use marijuana only in certain ways.” Participants were also asked to rate, on a scale of 0 to 100, how important it is to them to achieve their goal.

#### Depression

The PRISM (Hasin et al., 1996), a semi-structured interview with demonstrated reliability (Hasin et al., 1996) and validity for differentiating substance-induced psychiatric symptoms from psychiatric disorders that are temporally independent from substance use, was administered by a trained research assistant at baseline to assess for lifetime Major Depressive Disorder and to rule out Schizophrenia and Schizoaffective Disorder. The interviewer was trained to criterion on the PRISM using standardized procedures including didactic instruction, practice interviews, and direct observation.

The Patient Health Questionnaire (PHQ-9), a reliable and valid measure of depressive symptom severity (Spitzer, Kroenke, Williams, & Patient Health Questionnaire Primary Care Study Group 1999; Kroenke, Spitzer, & Williams, 2001) is a self-administered, 9-item questionnaire with scores ranging from 0 to 27. The PHQ-9 was administered at baseline and all subsequent visits during and after treatment.

#### Self-Efficacy

The Marijuana Self-efficacy Scale measured efficacy for avoiding use in situations involving negative affect, social discomfort, and presence of others using cannabis (Litt et al., 2005).

#### Coping Skills

The Coping Strategies Scale assesses use of CBT based coping skills. Ratings of frequency of use for various coping strategies provides a Total Coping score (Litt et al., 2005).

#### Health Related Quality of Life

The EQ-5D is a self-report, standardized instrument to measure health outcomes of five broad areas or domains: mobility, self-care, usual activities, pain/discomfort and anxiety/depression (Euroqol Group, 1990). Participants were asked to rate whether they have no problems, moderate problems or extreme problems as it pertains to each domain. EQ-5D descriptive scores were then converted to single summary index (Euroqol, 1990).

### Statistical Analysis

Cannabis use was indicated by: (1) number of cannabis-negative urine drug screens; (2) percentage of days using in the past 30 days; (2) number of times cannabis was used per day. Chi-square and paired t-tests were used to analyze baseline to post-treatment differences on key outcome variables. Subsequently, using pre- to post-treatment change scores, correlational analyses were employed to examine the association between changes in cannabis use and functional outcomes (e.g., depressive symptom severity, health-related quality of life).

## RESULTS

Treatment acceptability, defined as attending 2 or more urine testing appointments (greater than 1 week of study participation) was excellent (95%). On average, participants submitted 8.8 of 10 urine specimens over the course of treatment and attended 9.6 of the 10 SHADE treatment sessions, consistent with prior trials conducted in non-psychiatric settings. Treatment completion, defined as providing a urine specimen during Week 10, was achieved by the majority of participants (85%).

The percentage of days in the past 30 in which participants reported using cannabis declined from baseline (M=69%, SD=29%) to treatment-end (M=52%, SD=34%), an effect that approached significance, t(25)=1.9, p=0.05, *d* = 0.54. Nevertheless, the percentage of days of use was significantly lower than baseline at 1-month follow-up (M= 44%, SD=41%), t(22)= 2.3, p<0.05, *d* = 0.79. The number of times cannabis was used per day on use days also declined significantly from M=2.7 times per day (SD=1.6) at baseline to M=1.7 times at discharge (SD=0.9), t(22)=2.8, p<0.05, *d* = 0.77). At follow-up, the number of times cannabis was used per day remained lower than that observed before treatment initiation (M=2.1, SD=1.4); however, this difference was not statistically significant. Mean percentage of cannabis-negative toxicologies was 27% over the course of treatment, and 33% at 1-month follow-up. A little over a third of the sample (35%) achieved 2 or more consecutive weeks of abstinence from cannabis use over the 10-week intervention period.

In terms of cannabis treatment goals, 15% of participants reported that their objective was “to not use at all” and the remaining endorsed the following goal: “to use marijuana only in certain ways,” The average baseline rating for changing cannabis use as a goal was 53.5 (SD=26.3) out of 100, indicating that it was of moderate importance.

Depressive symptom severity was reduced from the moderately severe range at baseline (M=13.3, SD=4.7) to the mild range at week 10 (M=8.0, SD=5.3), t(23)=5.3, p<0.001, *d =* 0.89. At 1-month follow-up, severity was further reduced from baseline (m=6.0, SD=4.9), t(24)=7.3, p<0.001, *d =* 1.52.

Reductions in cannabis use were significantly correlated with changes in functional outcomes, including reductions in depressive symptoms at both treatment-end (r=0.41, p<0.05) and 1 month FU (r=0.51, p<0.05), and health-related quality of life, measured by the EQ-5D at treatment-end, albeit at the trend level (r=−0.35, p=0.12) and 1 month FU (r=−0,48, p<0.05).

Self-efficacy increased from baseline (M=65.5, SD=22.4) to treatment end (M=80.5, SD=31.2), t(21)=−3.03, p<0.01). Likewise, coping skills improved significantly from initiation to completion of treatment, t(22)=−3.2, p<0.01.

Perceived helpfulness of SHADE was assessed using a consumer feedback questionnaire. The majority of participants agreed or strongly agreed that: they liked the SHADE content overall (100%); the therapist check-in component of the intervention was helpful (90%); they learned new information about relapse, triggers, and cravings (70%); and the SHADE program helped them work towards their goals (90%). Qualitative participant feedback concerning SHADE corresponded with quantitative responses, and included the following: “SHADE gave me the tools. Before this I felt a lot more helpless about the situation, and this was something I would live with for the rest of my life, and when it happened [depressive episodes, cannabis use], I had no control. Now, I feel like... I have a lot more control over it.” SHADE homework “helped me focus throughout the week”, and the mindfulness activities “provided a conceptual framework for seeing the world from a more neutral perspective.”

To begin to examine costs associated with SHADE delivery, we measured the amount of therapist time spent on the clinician delivered component of the intervention at each session. On average, time spent face-to-face with a clinician was 13 minutes (SD=6) per session, with an overall total of 105 minutes (SD=31) over the course of the intervention.

## DISCUSSION

This is the first study to implement SHADE, a computer-assisted, integrated CUD and MDD intervention in a primary psychiatric care setting. Initial findings from this pilot investigation are promising, suggesting that introducing an integrated intervention in this setting is feasible and acceptable. A substantial proportion of screened participants who were receiving psychiatric care for MDD demonstrated interest in receiving an intervention targeting MDD and CUD concurrently, and among those who enrolled, the majority perceived the intervention as helpful. Likewise, most of the participants were retained in the computerized intervention, engagement and completion rates were excellent, reductions in depression were highly significant with corresponding large effect sizes, and changes in cannabis use were observed. Cannabis abstinence rates over the course of treatment, while somewhat lower than that observed in other psychosocial clinical trials that involved computerized or face-to-face CBT/MET (Budney et al., 2011), were nevertheless noteworthy given the variability in participants’ motivation to change their use of cannabis. Given the primary mental health care setting from which participants were recruited, it is not surprising that abstinence from cannabis use was infrequently endorsed as a treatment goal (i.e., only 15% reported an abstinence goal, with the remainder focused on reducing their use). Thus, although abstinence rates were somewhat lower in this study relative to prior trials among depressed cannabis users (Kay-Lambkin et al., 2009; 2011), the observed capacity of the SHADE intervention to facilitate reductions in cannabis use among individuals who were predominantly open to changing, but not abstaining from cannabis use, is encouraging. Likewise, despite the fact that cannabis use frequency at baseline was on average, somewhat lower than that reported in other clinical samples, changes in cannabis use, albeit moderate in terms of effect size, were observed from pre- to post-treatment.

The potential to use a computer to deliver evidence-based therapies targeting substance use among individuals with major depression has important implications for clinical practice. First, easily deployable strategies for treating substance use disorders outside of specialty addiction treatment settings are needed, particularly among those with multiple chronic conditions. Given the observation in this study that individuals in primary psychiatric care are receptive to an intervention addressing substance use, this model has the potential to be extended to other primary care settings, where substance use disorders are under recognized and undertreated, largely owing to the lack of availability of evidence-based treatments. Moreover, although the SHADE intervention includes clinician involvement to assess for safety and reinforce therapeutic skills training and practice, therapist involvement in the delivery of this intervention is quite minimal from a cost perspective, with an average of 10 minutes spent with each participant per session (total of 90 minutes over the course of the intervention). Although we did not conduct a cost analysis in the present study, prior work employing similar computerized CBT/MET intervention protocols among cannabis users has shown substantial cost savings associated with reduced therapist time (i.e., approximately 10 fold less time), when the computerized condition was compared to a face-to-face intervention of equivalent frequency (see Budney et al., 2011; 2015). Moreover, using computerized CBT as an adjunct to usual care is cost effective and of good value from both clinician and patient perspectives (Olmstead et al., 2010). Though computer purchase costs are incurred with computerized interventions, these costs are relatively minimal when compared to the personnel and space costs associated with face-to-face behavioral treatment delivery.

Another advantage of using computer-assisted interventions such as SHADE in the treatment of psychiatrically comorbid substance users is the potential reallocation of therapist time and effort for the most severe cases of multiple chronic conditions. Additionally, given that comorbid populations are often difficult to reach, schedule for treatment, retain, and follow, technology assisted approaches may be of great value and have not been extensively tested in complex populations with more than one Axis I disorder. Finally, the standardization of treatment content and delivery achieved through this medium enhances replicability as well as our ability to study and understand the components of treatment that are effective in improving target outcomes.

Despite the promise of our preliminary outcomes and the clinical practice implications of this novel approach to the treatment of co-occurring CUD and mood disorders, several limitations of the present investigation warrant comment. This study included a small sample size, did not employ control group for comparison with the SHADE intervention, and did not include post-treatment follow-up assessments beyond one month. Moreover, continuous abstinence rates may have been underestimated given the long half-life of cannabis and the corresponding latency for some individuals to produce a cannabis-negative urine sample. The lack of a control group raises perhaps the most critical issue. In the absence of a control condition, one cannot directly determine if the SHADE intervention produced positive outcomes or had no effect at all. However, two prior RCTs demonstrating the efficacy of the SHADE intervention among depressed cannabis users in reducing depressive symptoms and cannabis use (Kay-Lambkin et al., 2009; Kay-Lambkin et al., 2011), would suggest the former. Whether changes in cannabis use and depression in response to SHADE in a psychiatric treatment-seeking population are comparable to those achieved when CBT/MET are delivered face-to-face remains to be tested and is an important future direction for research into this approach. A planned randomized trial will address many of the limitations of the current pilot trial.

Although the present investigation provides only preliminary information concerning the potential efficacy of SHADE for individuals in a psychiatric setting with comorbid MDD and CUD, the results of this pilot study support prior data suggesting that computer-assisted interventions hold promise as a means of optimizing treatment service delivery for comorbid populations with substance use and mental health disorders. Technology-assisted platforms for the delivery of evidence-based interventions may address, in part, the multiple barriers to effective treatment of underserved populations with complex needs (e.g., need for integrated intervention content, limited availability of evidence-based approaches to comorbidity treatment). Future research addressing effective methods for implementation of these approaches both within and outside the health care system will facilitate accessibility of care for those with CUD and comorbid mental health disorders.
